# Trajectories of Postload Plasma Glucose in the Development of Type 2 Diabetes in Japanese Adults

**DOI:** 10.1155/2017/5307523

**Published:** 2017-09-14

**Authors:** Rie Oka, Kyoko Shibata, Masaru Sakurai, Mitsuhiro Kometani, Masakazu Yamagishi, Kenichi Yoshimura, Takashi Yoneda

**Affiliations:** ^1^Department of Internal Medicine, Hokuriku Central Hospital, Kanazawa University Hospital, Kanazawa, Japan; ^2^Department of Cardiovascular and Internal Medicine, Kanazawa University Graduate School of Medicine, Kanazawa, Japan; ^3^Department of Biostatistics, Innovative Clinical Research Center (iCREK), Kanazawa University Hospital, Kanazawa, Japan; ^4^Department of Social and Environmental Medicine, Kanazawa Medical University, Kanazawa, Japan

## Abstract

We aimed to clarify how the trajectories of 1-hour postload plasma glucose (PG) and 2-hour PG were different in the development of type 2 diabetes. Using data of repeated health checkups in Japanese workers from April 2006 to March 2016, longitudinal changes of fasting, 1-hour, and 2-hour PG on the oral glucose tolerance test were analyzed with a linear mixed effects model. Of the 1464 nondiabetic subjects at baseline, 112 subjects progressed to type 2 diabetes during the observation period (progressors). In progressors, 1-hour PG and 2-hour PG showed gradual increases with slopes of 1.33 ± 0.2 and 0.58 ± 0.2 mg/dL/year, respectively, followed by a steep increase by which they attained diabetes. Until immediately before the diabetes transition, age- and sex-adjusted mean level of 2-hour PG was 149 ± 2.7 mg/dL, 34 ± 2.7 (30%) higher compared to nonprogressors, while that of 1-hour PG was 206 ± 4.1 mg/dL, 60 ± 4.3 mg/dL (41%) higher compared to nonprogressors. In conclusion, diabetes transition was preceded by a mild elevation of 2-hour PG for several years or more. The elevation in 1-hour PG was larger than that of 2-hour PG until immediately before the transition to diabetes.

## 1. Introduction

Postload hyperglycemia is one of the early manifestations of impaired glucose metabolism. Conventionally, investigators defined postload hyperglycemia by 2-hour plasma glucose (PG) on the oral glucose tolerance test (OGTT) and demonstrated that the intervention at the stage of impaired glucose tolerance (IGT) was effective to prevent or delay the incidence of type 2 diabetes [1–3]. However, it has been recently reported that 1-hour PG has a superior predictive ability to 2-hour PG in three Western cohort studies [4–6] and in two Asian cohort studies [7, 8]. Cross-sectional investigations have also revealed that elevated 1-hour PG rather than 2-hour PG showed a stronger correlation with decreased insulin secretion assessed by insulinogenic index [4, 5, 7] or acute insulin response on the intravenous glucose tolerance test (IVGTT) [9]. The measurement of 1-hour PG cannot be ignored to detect the individuals with impairment of postload glucose metabolism.

In a few studies on trajectories of postload PG in the development of type 2 diabetes, 2-hour PG showed a gradual linear increase for more than 10 years, followed by a rapid deterioration before the diagnosis [10–12]. They reported that 2-hour PG on average was regulated within the normal range (<140 mg/dL) until 2 years before the onset of diabetes [10, 12]. They and any other investigators have not reported the trajectory of 1-hour PG. We hypothesized that 1-hour PG might increase earlier than 2-hour PG in those who later developed diabetes, which made its predictive ability better.

The aim of this study, therefore, was to depict trajectories of postload PG levels during the development of type 2 diabetes, with an interest in the difference of 1-hour PG from 2-hour PG. The optimal cutoff point of 1-hour PG in this population was also estimated.

## 2. Methods

### 2.1. Study Subjects

**Figure 1:**
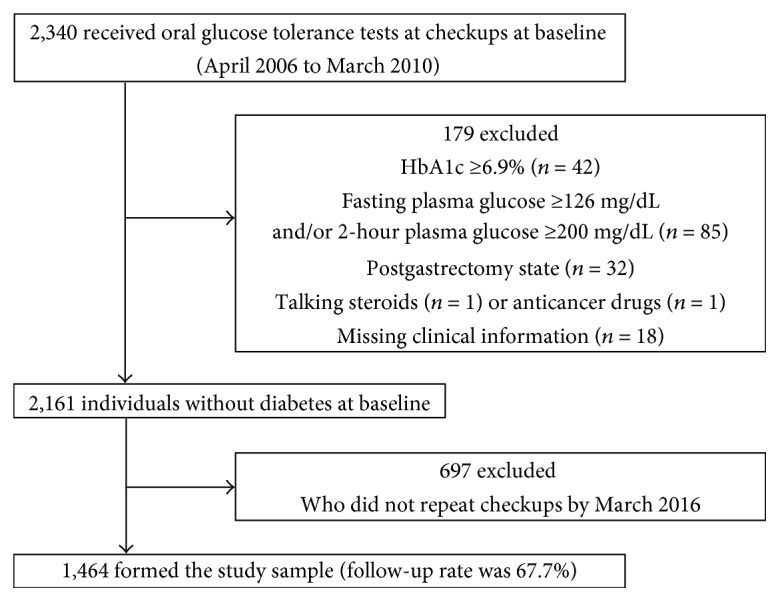
Study sample.

A historical cohort study was conducted using data from the medical checkups of public school employees collected in the Hokuriku Central Hospital [7]. During April 2006 and March 2010 (baseline period), 2340 employees underwent an OGTT at a checkup [13]. If employees received more than one checkup during the baseline period, the initial checkup data were used. After those who had fasting PG ≥ 126 mg/dL and/or 2-hour PG ≥ 200 mg/dL (*n* = 85), who had HbA1c values ≥6.9% (52 mmol/mol) (*n* = 42), who had undergone gastrectomy (*n* = 32), who were taking steroids (*n* = 1), who were taking anticancer drugs (*n* = 1), or who had any missing data (*n* = 18) were excluded, we selected 2161 nondiabetic individuals. Of the 2161 thus selected, 1464 individuals repeated checkups at least once by March 2016 and comprised our study sample (Figure 1). The subjects were followed until they developed diabetes; if they remained free of diabetes, follow-up ended at the time of the last checkup. The remaining 697 subjects did not repeat checkups, which meant a follow-up rate of 67.7%. An OGTT was performed at all checkups during the follow-up period; but in the last two years (from April 2014 to March 2016), an OGTT was performed on 1191 subjects and the measurement of fasting PG only was performed in the remaining 273 subjects due to the financial reason of the mutual aid association. Information on smoking and drinking habits and medical history was obtained through a questionnaire. Subjects were considered current smokers if they smoked at least one cigarette per day. Alcohol use was defined by the number of days per week for drinking regardless of the amount consumed. Signed informed consent was obtained from all subjects, and the hospital review board approved the study protocol. The study was registered on the University Hospital Medical Information Network Clinical Trials Registry (UMIN-CTR, UMIN ID: UMIN000017662).

### 2.2. Diagnosis of Diabetes and Blood Sampling

Diabetes was diagnosed if fasting PG ≥ 126 mg/dL, 2-hour PG ≥ 200 mg/dL, and/or receiving treatments for type 2 diabetes based on the World Health Organization criteria (WHO) [14]. All the evaluations were performed at the health check department of the Hokuriku Central Hospital. Subjects were asked to visit the hospital between 8:00 a.m. and 9:00 a.m. after an overnight fast. At the baseline visit, an OGTT (75 g dextrose monohydrate in 250 ml water) with 0, 30, 60, and 120 min sampling to determine PG and insulin levels was performed on all subjects [13]. HbA1c assay was conducted at the central clinical laboratory in our hospital by a high-performance liquid chromatography (HPLC) method using ADAMS HA-8170 (ARKRAY, Kyoto). The following indices of insulin secretion and insulin sensitivity were calculated in this study: insulinogenic index = (Ins_30_ − Ins_0_[mU/L])/(Gluc_30_ − Gluc_0_[mg/dL]), where Ins_y_ and Glu_y_ represent values at time *y* min during the OGTT [15] and homeostatic model assessment of insulin resistance (HOMA) = Glu_0_(mg/dL) × Ins_0_(mU/L)/405 [16].

### 2.3. Statistical Analysis

Analyses were conducted using SPSS software version 17.0 for Windows (SPSS Inc. Chicago, USA). Data are presented as the mean ± SD or the median with the interquartile range for continuous variables or as a frequency for categorical variables. Subjects were divided into two groups, those who progressed to diabetes (progressors) and those who did not (nonprogressors). The differences in the baseline characteristics were tested using Student's *t*-test or Mann–Whitney *U* tests for continuous variables or *χ*^2^ test for categorical values. The mixed effects model was used to estimate the trajectories of fasting PG, 1-hour PG, and 2-hour PG [17]; this technique takes into account within-subject correlations from repeated measurements with unequal numbers of observations per subjects and unequal intervals between measurements. As fixed effects, we entered time with sex and age as covariates into the model. We set a timeline with 0 year at the year of diagnosis for progressors or at the last checkup for nonprogressors, and time of prior measurements was coded backward, giving minus values. As a random effect, we included the participant's identification number. We used SPSS to calculate the annual estimated levels of PG and used *R* to fit the model explaining the PG trajectories. The difference of the slopes of increase by time between progressors and nonprogressors was examined by the interaction between a dummy variable for progression (0 = nonprogressors; 1 = progressors) and time in the model. Finally, to identify the optimal cutoff point for progression of type 2 diabetes, subjects were divided by their baseline data at 140 mg/dL of 2-hour PG based on the cutoff value for IGT [14], at 155 mg/dL of 1-hour PG based on the prior study by Abdul-Ghani et al. [18], and at 180 mg/dL of 1-hour PG based on the committee report of Japan Diabetes Society [19]. Then the hazard ratio (HR) of the development of diabetes of those over versus those below the cutoff point was calculated by Cox proportional hazard regression analysis. The HR was calculated using three models: Model 1, the multivariable-adjusted model, with covariates including age, sex, BMI, fasting PG, HbA1c, smoking status (three-level variable: current/former/never smoker), alcohol use (three-level variable: drinking everyday/drinking 1–6 days per week/drinking less than 1 day per week), taking antihypertensive drugs (yes or no), and taking lipid-lowering drugs (yes or no); Model 2, in which Model 1 was additionally adjusted for the other PG values (i.e., 2-hour PG value for the division by 1-hour PG and 1-hour PG value for the division by 2-hour PG); and Model 3, in which Model 2 was further adjusted for insulinogenic index and HOMA index. A *p* value <0.05 was considered statistically significant.

## 3. Results

### 3.1. Characteristics of Subjects

The study subjects were composed of 1464 subjects with a mean age of 52.0 ± 7.0 years and a mean BMI of 23.9 ± 3.2 kg/m^2^ at baseline. Of these, 112 subjects developed type 2 diabetes during the observation, diagnosed by fasting PG ≥ 126 mg/dL (*n* = 51), 2-hour PG ≥ 200 mg/dL (*n* = 72), and/or receiving treatments for type 2 diabetes (*n* = 2). The remaining 1352 subjects stayed free of diabetes until follow-up.  Table 1 presents the baseline characteristics of the two study groups: progressors and nonprogressors. Progressors included a higher proportion of men and had higher BMI, higher PG and insulin concentrations during OGTT, higher HbA1c, higher HOMA index, and lower insulinogenic index compared with nonprogressors (*p* < 0.05). Because 75 subjects had insulinogenic index values ≤0, the analysis including insulinogenic index was performed on 1389 subjects.

### 3.2. Mixed Effects Model Analysis

**Figure 2:**
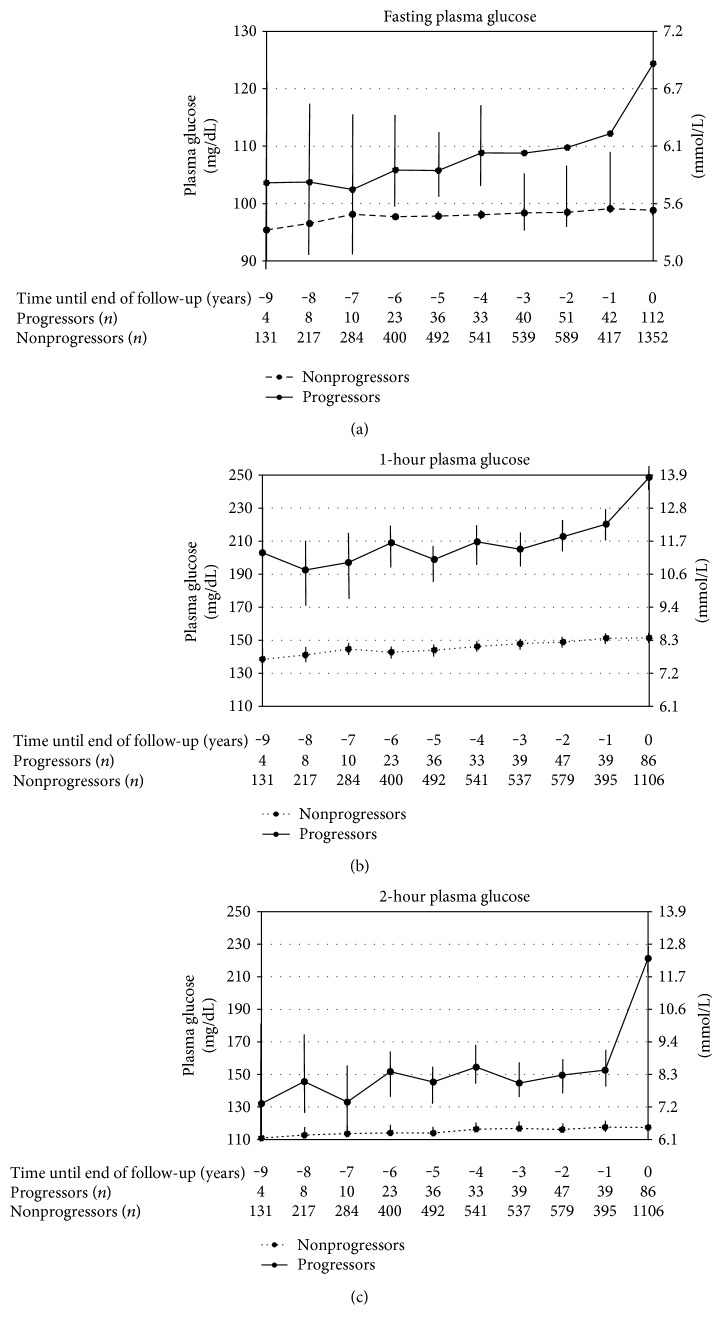
Trajectories of fasting (a), 1-hour (b), and 2-hour (c) plasma glucose until the incidence of type 2 diabetes in 112 progressors compared to 1352 nonprogressors. Analyses were adjusted for age and sex in mixed effects models. Error bars show 95% confidence intervals for the estimated levels.

A mixed effects model was used as this enables effective use of repeated measurements even when numbers and intervals of observations were different per subjects. The number of observations in each year was shown at the bottom of Figure 2. Fasting PG in nonprogressors showed a slight increase from 95 ± 0.5 mg/dL to 98 ± 0.2 mg/dL during the 9 years of observation. In progressors, fasting PG showed a gradual increase until >1 year, followed by a steep increase immediately before the diagnosis (from 112 ± 2.6 to 124 ± 1.7 mg/dL) (Figure 2(a)). A steep increase immediately before the diagnosis in progressors was also observed for 1-hour PG (from 220 ± 5.4 to 249 ± 3.9 mg/dL) (Figure 2(b)) and for 2-hour PG (from 155 ± 5.4 to 221 ± 3.9 mg/dL) (Figure 2(c)). Compared to >9 years, the estimated level was not significantly different in any other year but 0 year for 1-hour PG (*p* < 0.01) and for 2-hour PG (*p* < 0.001). Age- and sex-adjusted mean level of 1-hour PG until >1 year was 206 ± 4.1 mg/dL, 60 ± 4.3 mg/dL (41%) higher compared to nonprogressors (*p* < 0.001), and that of 2-hour PG was 149 ± 2.7 mg/dL, 34 ± 2.7 mg/dL (30%) higher compared to nonprogressors (*p* < 0.001). In 112 progressors, between the final and prefinal measurements of glucose concentrations, 42 (38.5%) had a 1-year interval, 31 (28.4%) had a 2-year interval, and the remaining 39 (33.1%) had a 3 years or more interval, with a median value being 2 years in all progressors. When mixed effects model analysis was conducted in 28 progressors whose annual consecutive data were available until the diagnosis of diabetes, steep increases in 1-hour PG and 2-hour PG were also demonstrated (Supplementary Figure available online at https://doi.org/10.1155/2017/5307523).

To test this statistically, we fit a model including time as a continuous variable plus a dummy variable for 0 year (1 = 0 year; 0 = from −9 to −1 year). The model including this dummy variable (piecewise model) fits better than the model with only continuous time variable (linear model) as assessed by Akaike information criterion (AIC). As shown in Table 2, large regression coefficients for progression (49.9 for 1-hour PG and 29.0 for 2-hour PG) indicated the difference between progressors and nonprogressors throughout the observation. The interaction term progression × 0 year but not progression × time was significant both for 1-hour and 2-hour PG, indicating that the slope of increase was not significantly different between progressors and nonprogressors until >1 year. When diabetes was defined only by fasting PG ≥ 126 mg/dL and/or receiving treatments for type 2 diabetes; that is, individuals with isolated postload hyperglycemia were excluded from progressors, the results were similar (data not shown).

### 3.3. Predictive Abilities of Different Cutoff Points for 1-Hour PG and 2-Hour PG

Table 3 shows the HR for the development of diabetes of those over versus those below the three different cutoff points. The HR of those with 1-hour PG ≥ 155 mg/dL versus 1-hour PG< 155 mg/dL and the HR of those with 1-hour PG ≥ 180 mg/dL versus 1-hour PG< 180 mg/dL were significantly elevated even after adjusted for 2-hour PG, insulinogenic index, and HOMA index. The elevated HR of those with 2-hour PG ≥ 140 mg/dL versus 2-hour PG< 140 mg/dL was significant (*p* < 0.05) but was attenuated after further adjustments for 1-hour PG.

## 4. Discussion

In this study, we depicted trajectories of postload plasma glucoses during the development of type 2 diabetes in Japanese workers. In agreement with prior studies [10–12], diabetes transition was preceded by a mild elevation of 2-hour PG for many years where the slopes of increase were not significantly different between progressors and nonprogressors. The difference between progressors and nonprogressors was relatively large in 1-hour PG rather than 2-hour PG until immediately before the diagnosis. These trajectories help to understand why 1-hour PG better predicts the future development of type 2 diabetes than 2-hour PG.

A rapid glucose rise in 2-hour PG immediately before the onset of diabetes (from −1 to 0 year) confirmed in this study was consistent with several cohort studies [10–12, 20, 21]. In the Pima longitudinal study which comprised 20 years before the development of diabetes, an exponential increase in the 2-hour glucose levels occurred during the final 4 years preceding the onset [10]. Ferrannini et al. also reported that the natural history of diabetes was marked by a steep increase in 2-hour plasma glucose in 3 years or shorter time frame, in which they discussed instability paradigm, in individuals who are destined to become diabetic; insulin sensitivity and insulin secretion are more or less altered, generating a critical state of instability [11]. In such an unstable condition, a relatively small further change would result in a large, rapid rise in glucose concentrations. In the Whitehall II Study, the magnitude of this rapid increase in 2-hour PG was larger than 70 mg/dL from >2 to 0 year [12], which was comparable to ours (68 mg/dL from >1 to 0 year). As diabetes was attained by such a rapid rise in the short time frame, the difference of 2-hour PG between progressors and nonprogressors does not become large until just before the diagnosis.

Mild elevation in 2-hour PG levels continued for several years or more until just before the aforementioned rapid rise. During this period, the slope in 2-hour PG as well as 1-hour PG was not significantly different between progressors and nonprogressors, in agreement with the results from the Whitehall II Study [12]. Therefore, contrary to our hypothesis, 1-hour PG did not increase earlier or faster than 2-hour PG, but the difference between progressors and nonprogressors present at the commencement of the observation was maintained for many years. As the difference in 1-hour PG was relatively larger rather than 2-hour PG until immediately before the diagnosis, it would be natural that progressors and nonprogressors were better discriminated by 1-hour PG than 2-hour PG.

Higher 1-hour PG than 2-hour PG is common in nondiabetic people as demonstrated by the studies on the shape of glucose curve during the OGTT [22–24]; about half of the study subjects had monophasic glucose curves, with a peak during 30–90 min followed by a decrease during 90–120 min. The second popular type was biphasic, who had a nadir by 90 min and an increase again during 90–120 min. Compared to subjects with biphasic shape, monophasic subjects had a worse insulin sensitivity, lower insulin secretion, higher 1-hour PG [22–24], and higher risk for type 2 diabetes [25]. The elevation of 1-hour PG might reflect the characteristic of these monophasic type of subjects. Whether the elevation of 1-hour PG is acquired or inherent is not known, but elevated 1-hour PG is likely to be present in the very early years in the natural course of the development of type 2 diabetes.

The cutoff point of 155 mg/dL for 1-hour PG was first proposed by Abdul-Ghani et al. [18] and has been demonstrated to be useful to identify the individuals at higher risk for type 2 diabetes in other independent cohort studies from Finnish [5], Jewish [8], and Asian Indian populations [26]. Abdul-Ghani et al. set 155 mg/dL based on the result that the sum of sensitivity and specificity was maximal (0.75 and 0.79 for sensitivity and specificity, resp.), and they also reported that the optimal cutoff point for fasting PG was as low as 94 mg/dL applying the same method [18]. Indeed, the HR for future diabetes was significantly elevated both in NGT and IGT with 1-hour PG 155 ≤ mg/dL in this study. However, 44% of our study subjects had 1-hour PG 155 ≤ mg/dL at baseline. Such a lower cutoff point might bring problems with socioeconomic costs as the new definition of “prediabetes” did so [27, 28]. As Asians have greater glycaemic excursions after taking glucose than Europeans [29], the ethnicity may also be taken into account to set the optimal cutoff point for 1-hour PG.

The strength of our study is the longitudinal observation of postload plasma glucoses, which allow for the comparison between the trajectory of 1-hour and that of 2-hour PG. However, several limitations of this study should be considered. First, data on subjects who did not receive checkups during the follow-up period were not available. Subjects who missed checkups might be less conscious about their health, which biased the study subjects toward metabolically healthy people. However, baseline characteristics including BMI, glucose, and insulin concentrations between the subjects who were followed and those who were missed were not significantly different. Second, 60 out of 112 progressors were diagnosed as diabetes only by 2-hour PG 200 ≤ mg/dL with normal fasting PG (<126 mg/dL), and the estimated value of fasting PG at the diagnosis (0 year) in progressors was relatively low compared to the prior studies. Our results may reflect more the character of isolated postload hyperglycemia rather than combined fasting and postload hyperglycemia. However, when diagnosis of diabetes was conducted only by fasting PG and/or receiving treatments, the results were similar. Third, the measurement of PG concentrations relied on a single OGTT, which is known to have within-subject variability [30].

In conclusion, longitudinally observed, diabetes transition was preceded by a mild elevation of 2-hour PG for many years. The difference in 1-hour PG between progressors and nonprogressors was relatively large until immediately before the diagnosis.

## Supplementary Material

Supplementary Figure. Trajectories of fasting (A), 1-hour (B), and 2-hour (C) plasma glucose until the incidence of type 2 diabetes in 28 progressers whose annual consecutive data were available. Error bars show 95% confidence intervals for the estimated levels after adjustments for age and sex.

## Figures and Tables

**Table 1:** Baseline characteristics of the subjects.

	Nonprogressors	Progressors
*n*	1352	112
Age (years)	52 ± 7	53 ± 7
Sex (% male)	69	79^∗^
Body mass index (kg/m^2^)	23.8 ± 3.1	25.2 ± 3.7^∗^
Fasting plasma glucose (mg/dL)	95 ± 7	108 ± 9^∗^
30 min plasma glucose (mg/dL)	149 ± 29	185 ± 29^∗^
1-hour plasma glucose (mg/dL)	144 ± 41	209 ± 32^∗^
2-hour plasma glucose (mg/dL)	113 ± 25	148 ± 31^∗^
Fasting insulin (mU/L)	2.8/3.8/5.1	2.9/4.3/7.2^∗^
30 min insulin (mU/L)	19.2/28.6/45.2	12.5/22.7/35.3^∗^
HbA1c (%)	5.0/5.2/5.4	5.4/5.6/5.9^∗^
Insulinogenic index (*n* = 1389)	0.30/0.50/0.88	0.14/0.24/0.43^∗^
HOMA index	0.66/0.90/1.26	0.80/1.11/1.98^∗^
Current smokers (%)	19.9	26.8^∗^
Drinkers (%)	64.3	59.8
Antihypertensive medications (%)	13.2	19.6^∗^
Lipid-lowering medications (%)	6.8	8.0

Data are expressed as mean ± SD, 25/50/75th percentile value, or number (%). ^∗^*p* < 0.05. HOMA: homeostasis model assessment of insulin resistance.

**Table 2:** Fixed effects in the mixed effects models for changes of fasting, 1-hour, and 2-hour plasma glucose values in 112 progressors compared to 1352 nonprogressors.

	Fasting PG (mg/dL)		1-hour PG (mg/dL)		2-hour PG (mg/dL)	
	Regression coefficient	SE	Regression coefficient	SE	Regression coefficient	SE
Intercept	89.9	0.9	105.2	4.2	101.6	2.7
Time (per year)	0.22	0.0	1.33	0.2	0.58	0.2
Progression	—	—	49.9	7.9	29.0	5.7
Progression × time	1.23	0.2	—	—	—	—
Progression × year 0	10.1	1.1	28.3	4.9	67.6	3.9

Piecewise mixed effects modeling adjusted for age and sex. Time = a continuous variable set at the year of diagnosis for progressors or at the last checkup for nonprogressors with 0; progression = a dummy variable, 1 for progressors and 0 for nonprogressors; all coefficients are significant, *p* < 0.001.

**Table 3:** Hazard ratios (HR) for the development of diabetes by different cutoff points of 1-hour PG and 2-hour PG.

Cutoff point	Sensitivity (%)	Specificity (%)	Crude HR (95% CI)	Adjusted HR (95% CI)		
				Model 1	Model 2	Model 3
1-hour PG ≥155 mg/dL (8.6 mmol/L) (yes versus no)	92.9	60.3	18.2 (8.9–37.4)	7.7 (3.6–16.3)	5.9 (2.7–12.7)	5.1 (2.2–11.8)
1-hour PG ≥180 mg/dL (10.0 mmol/L) (yes versus no)	83.0	78.3	15.9 (9.7–26.0)	6.8 (3.9–11.9)	5.2 (2.9–9.4)	4.3 (2.3–8.1)
2-hour PG ≥140 mg/dL (7.8 mmol/L) (yes versus no)	60.7	83.4	7.3 (5.0–10.8)	3.1 (2.0–4.8)	1.6 (1.0–2.6)	1.6 (1.0–2.6)

Model 1: adjusted for age, sex, body mass index, fasting PG, HbA1c, smoking status, alcohol use, taking antihypertensive drugs, and taking lipid-lowering drugs. Model 2: adjusted for Model 1 variables plus the other PG (i.e., 2-hour PG for cutoff of 1-hour PG and 1-hour PG for cutoff of 2-hour PG). Model 3: adjusted for Model 2 variables plus insulinogenic index and HOMA index.
