# Supplementary material for: Trajectories of Postload Plasma Glucose in the Development of Type 2 Diabetes in Japanese Adults
Source: J Diabetes Res. 2017 Sep 14;2017:5307523. doi: 10.1155/2017/5307523 (PMC5618754; doi:10.1155/2017/5307523)
Supplement: Supplementary file 1 — Supplementary Figure. Trajectories of fasting (A), 1-hour (B), and 2-hour (C) plasma glucose until the incidence of type 2 diabetes in 28 progressers whose annual consecutive data were available. Error bars show 95% confidence intervals for the estimated levels after adjustments for age and sex. [file 5307523.f1.docx]

**Supplementary Figure.**

Trajectories of fasting (A), 1-hour (B), and 2-hour (C) plasma glucose until the incidence of type 2 diabetes in 28 progressers whose annual consecutive data were available. Error bars show 95% confidence intervals for the estimated levels after adjustments for age and sex.

**(A)**

　　　　　
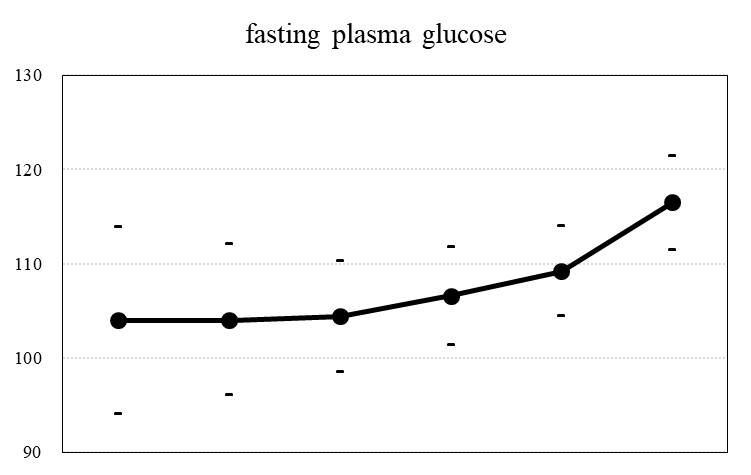


**plasma glucose**

(mg/dL)

| **Time until end of follow-up (years)　-5 -4 -3 -2 -1 0** |
| --- |
| **Progressors (n) 3 5 12 19 28 28** |

**
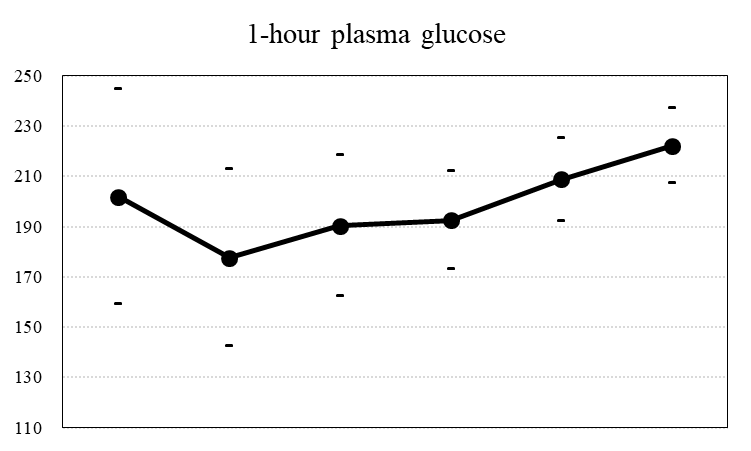
(B)**

(mg/dL)

| **Time until end of follow-up (years)　-5 -4 -3 -2 -1 0** |
| --- |
| **Progressors (n) 3 5 12 19 28 28**  **(C)**   \| **Time until end of follow-up (years)　-5 -4 -3 -2 -1 0** \| \| --- \| \| **Progressors (n) 3 5 12 19 28 28** \|   **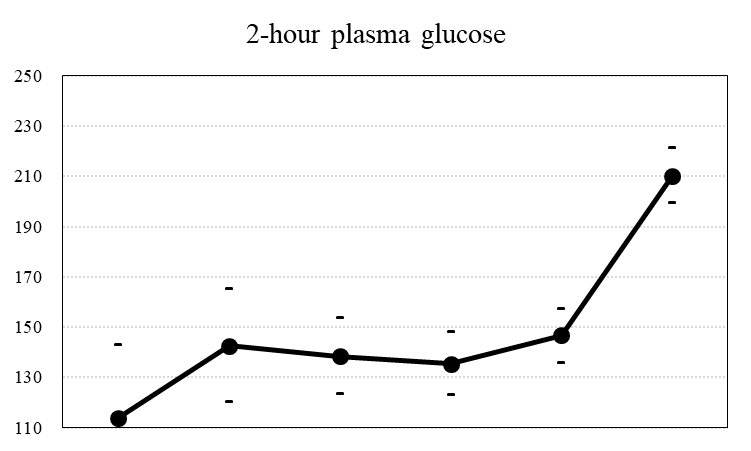**  **plasma glucose**  (mg/dL) |

| **plasma glucose** |  |  |  |  |  |  |  |  |  |  |
| --- | --- | --- | --- | --- | --- | --- | --- | --- | --- | --- |
|  |  |  |  |  |  |  |  |  |  |  |
|  |  |  |  |  |  |  |  |  |  |  |
